# Safety Study of Combination Treatment: Deep Brain Stimulation and Transcranial Magnetic Stimulation

**DOI:** 10.3389/fnhum.2020.00123

**Published:** 2020-04-03

**Authors:** Hamzah Magsood, Farheen Syeda, Kathryn Holloway, Ivan C. Carmona, Ravi L. Hadimani

**Affiliations:** ^1^Department of Mechanical and Nuclear Engineering, Virginia Commonwealth University, Richmond, VA, United States; ^2^Department of Biomedical Engineering, Virginia Commonwealth University, Richmond, VA, United States; ^3^McGuire Research Institute, Hunter Holmes McGuire Veterans Affairs (VA) Medical Center, Richmond, VA, United States; ^4^Department of Neurosurgery, Virginia Commonwealth University Health System, Richmond, VA, United States

**Keywords:** DBS, TMS, Parkinson’s, safety study, combined treatment, experimental, head models

## Abstract

Patients with advanced Parkinson’s disease (PD) often receive deep brain stimulation (DBS) treatment, in which conductive leads are surgically implanted in the brain. While DBS treats tremor and rigidity, patients often continue to suffer from speech and swallowing impairments. There is preliminary evidence that transcranial magnetic stimulation (TMS) of the cortex may be beneficial for these symptoms. However, the potential electromagnetic interactions of the strong magnetic fields from TMS on the conductive leads is unknown, and the combination therapy has not been approved for use. In this article, we report an experimental study of the safety of combining DBS and TMS. We fabricated an anatomically accurate head and brain phantom with electrical conductivities matching cerebrospinal fluid and averaged conductivity of gray and white matter. Induced current on an implanted DBS probe in the brain phantom was measured. Our results show that TMS will induce current values in the range or higher than typical DBS stimulation current. Thus, the combination of TMS/DBS treatment might cause over-stimulation in the brain when stimulated directly over the DBS lead with 100% TMS current intensity.

## Introduction

Patients with Parkinson’s disease (PD) suffer from debilitating symptoms, including bradykinesia, resting tremor, shuffling gait, and rigidity as well as hypophonic speech and swallowing difficulties (Hartelius and Svensson, 1994; Chaudhuri et al., 2006; Jankovic, 2008). Although the symptoms initially respond to Levodopa, a dopaminergic medication, the symptoms often become refractory or side effects of the medications can cause additional symptoms (Marsden and Parkes, 1976, 1977; Melamed, 1979). In these cases, physicians recommend deep brain stimulation (DBS) surgery, in which one or two electrical leads are inserted into the subthalamic nucleus (STN) or globus pallidus internus (GPi), and an electrical current is continuously delivered to these nuclei from a battery pack inserted into a chest pocket as it is on an “On” state (Perlmutter and Mink, 2006; Coffey, 2009; Lozano and Lipsman, 2013). DBS has been shown to effectively improve motor symptoms in patients of both PD as well as essential tremor (Koller et al., 2001; Perlmutter and Mink, 2006; Coffey, 2009). However, one of the more crippling symptoms of PD that is not treated by DBS is hypophonic speech and swallowing difficulty (dysphagia). Not surprisingly, hypophonia and dysphagia can seriously deteriorate the quality of life and cause complications such as weight loss, isolation, and depression (Schrag et al., 2000; Plowman-Prine et al., 2009; Crary et al., 2012). Importantly, these symptoms typically present well after the other motor symptoms have become problematic, thus most patients with speech and swallowing symptoms will have received DBS by the time of onset. The mouth motor area of the primary motor cortex is thought to play a role in the pathophysiology of these symptoms, and manipulation of this cortex through repetitive Transcranial Magnetic Stimulation (rTMS) has been proposed as a treatment option (Hartelius and Svensson, 1994; Pascual-Leone et al., 1994, 1995; Fox et al., 2001; Blank et al., 2002; Kobayashi and Pascual-Leone, 2003). rTMS is a non-invasive neuromodulation therapy that utilizes time-varying magnetic fields to induce electric fields in the patient’s brain, thus stimulating neurons in the targeted region (George et al., 1999, 2000). However, the potential for electromagnetic interference from the magnetic fields of rTMS with the conductive leads of DBS has precluded clinical implementation of the combination therapy. Primarily, there is concern regarding eddy currents, which are induced on conductive surfaces caused by time-varying magnetic fields. It is hypothesized that B-fields from rTMS may induce such currents on the surface of any conductive part of the lead, and this current would travel along the lead down to the contacts, in turn stimulating the deep brain nuclei which the contacts target. We posit that this issue has yet to be studied with accurate models and parameters. In the past, studies have considered the implications of combining DBS with rTMS, but we argue that these studies are quite limited. Some have underestimated and oversimplified the geometrical complexities of the lead and biological tissue (Deng et al., 2010). Besides, we are not aware of any studies as of yet which have used physical models with accurate head and brain geometry and impedances to study the effects of TMS on full implanted DBS leads (Kumar et al., 1999; Deng et al., 2010; Shimojima et al., 2010; Kühn and Huebl, 2011). The consideration of the accurate impedances and geometries account for any energy couplings of displacement currents with the induced current in the DBS lead. A brief literature review outlining differences between these publications and our present work, including general methods and findings, is presented in Table 1.

**Table 1:** Previous publications studying the safety of combination transcranial magnetic stimulation/deep brain stimulation (TMS/DBS) treatments.

Publication	Methods	Current Induced in DBS Leads	Results/Notes
Kumar et al. (1999)	Homogeneous phantom head model. TMS at 100% intensity performed 1 cm above leads. Voltage measured between contacts.	70–125 μA	Induced current lower than DBS stimulation.
Shimojima et al. (2010)	Homogeneous phantom head model. TMS applied at various locations along head model Impedance used: 1,162 Ω	>20 μC/cm^2^/phase	Current too high for stimulation to be performed safely.
Kühn and Huebl (2011)	TMS at 100% intensity with DBS ON at 4V. Voltage measured between contacts.	0.2–2.8 V	Voltage does not exceed DBS stimulation and TMS duration is too short to cause stimulation.
Deng et al. (2010)	Created full circuit from contacts to chest IPG. Did not use full DBS lead geometry.1.2 kΩ resistor with contacts.	Up to 83 mA. If DBS is OFF, current is only possible at V >5V.	Induced current too high for stimulation to perform safely.
Kühn et al. (2002)	Clinical investigation of five patients with bilateral DBS and TMS in the Motor Cortex.	N/A	Contralateral and ipsilateral motor-evoked potentials were induced in 3/5 patients from TMS. No other complications reported.
Hidding et al. (2006)	Clinical investigation of eight Parkinson’s patients with DBS, and mono pulse TMS in the Motor Cortex.	N/A	MEP latencies were significantly shortened, possibly due to current induced from TMS. No other complications reported.
Current work	Anatomically accurate head phantom. TMS at 100% intensity	(1.71–3.20 mA)	Induced current higher than DBS stimulation.

We have previously studied the effects of TMS on a conductive cylinder and individual lead contacts in deep brain regions. We focused on the E-field induced in the brain tissues surrounding the conductive probe and found that although there was a slight increase in E-field in the tissues surrounding the lead, E-field values did not come close to the stimulation threshold (Syeda et al., 2017). However, that study did not include the geometrical complexities of DBS wires within the lead; these model details would enable a more comprehensive study of TMS-induced current inside the lead body. While E-field at the contact locations may not have reached the stimulation threshold, it is crucial to determine the current induced in the conductive wires, as this current would potentially lead to deep brain stimulation. In our previous study, we did not consider a closed loop and the simulation was applied at motor threshold instead of 100% TMS power. In this article, we report an experimental study of the safety of combining DBS and TMS in a realistic head phantom. We fabricated an anatomically accurate head and brain phantom based on MRI images developed into the brain model using FreeSurfer, simNIBS, and FSL pipelines. The head phantom is fabricated with electrical conductivities matching cerebrospinal fluid and averaged conductivity of gray and white matter. Induced current on an implanted DBS probe in the brain phantom was measured.

rTMS is currently FDA-approved for the treatment of a drug-resistant major depressive disorder but has shown beneficial effects for symptoms of other neurological conditions (George et al., 1999; Klein et al., 1999; Wassermann and Lisanby, 2001; Khedr et al., 2005). During TMS, alternating current is run through a figure-of-8 coil, which causes a time-varying magnetic field. This B-field then propagates through the patient’s skull and onto the brain cortex, where an electric field is induced and neurons in the targeted cortical region are depolarized (Chen et al., 1997; George et al., 1999; Wassermann and Lisanby, 2001). rTMS in the mouth area of the primary motor cortex may help relieve hypophonia and dysphagia by similarly induced plasticity (Pascual-Leone et al., 1994, 1995; Kobayashi and Pascual-Leone, 2003).

Because TMS induces a time-varying magnetic field, it is important to consider the effects of TMS on conductive DBS leads. From Faraday’s law of induction, it is clear that any time-varying magnetic field will induce an electric field on a conductive substance and a subsequent eddy currents on the conductive surfaces of the lead wires. In this study, we explore factors that mediate the intensity of this current. Furthermore, the location of the lead tip is typically at similar sites in each patient, at the GPi or STN. However, it is also important to note that the course of the additional lead body lying on the surface of the skull is closest to the TMS coil. Therefore, it is important to consider the distance between the lead and TMS coil.

## Materials and Methods

Generally, clinicians use either bipolar configuration or unipolar configuration of DBS. In the bipolar configuration, the current is delivered to one contact such that the voltage difference between the contact and its neighboring contact creates a sphere of potential at the stimulation location. In the unipolar configuration, the generator case is positive and a single contact is negative (McIntyre et al., 2004; Butson and McIntyre, 2005; Wei and Grill, 2005). The current induced in either of these configurations, on the order of mA, must be compared against current-induced due to TMS B- fields. Therefore, we have tested these leads in the “OFF” position, with no direct current applied to the leads. Moreover, we have assumed that the surgeon has tunneled the lead directly posteriorly towards the vertex so that the subgaleal lead is relatively medial, while the TMS coils are placed laterally and inferiorly to access the ipsilateral mouth motor cortex.

### Anatomically Realistic Head Phantom With Implanted DBS Preparation

We fabricated an anatomically accurate head/brain phantom with an implanted DBS probe at the hypothetical STN location. The head model consists of four main parts: (1) the brain phantom; (2) skull, skin, and scalp; (3) cerebrospinal fluid; and (4) implanted DBS probe. The process of creating the brain and head phantom can be found in detail in our published patent where we show the detailed steps for creating each part of the head phantom (Magsood et al., 2019; Magsood and Hadimani, Under Review). In the following sections, we briefly show a general procedure to obtain each part of the head phantom with an implanted DBS.

### Brain Phantom

The brain phantom was fabricated using a healthy subject’s MRI images downloaded from the online database of human connectome project HCP, Parkinson’s Progression Marker Initiative (PPMI; Human Connectome Project HCP, 2018). The MRIs were segmented and a 3D brain model was developed into an stl format using FreeSurfer (Athinoula A. Martinos Center for Biomedical Imaging, Charlestown, MA, United States), SimNIBS (Danish Research Centre for Magnetic Resonance, DRCMR), and FSL (Analysis Group, Oxford, UK) software. From the model, we created shells to serve as molds for the segments of the brain. For example, to create the gray matter we used the head model and created the outer shells of the gray matter. These shells were 3-D printed and were used as molds for the constituent material of the brain phantom. A conductive polymer composite was prepared to mimic the electrical conductivity of the brain. The conductive polymer composite is composed of multi-walled carbon nanotubes (MWCNT) and polydimethylsiloxane (PDMS). The addition of the MWCNT to the PDMS imparts an electrical conductivity depending on the concentration of the MWCNT. The conductive polymer is poured into the molds and left to solidify. After the solidification, the molds are immersed in acetone to be removed and to obtain the accurate anatomy of the brain matching the MRI. The brain phantom was fabricated with a measured impedance of 450–500 Ω that matches the average impedance of the human brain (Gabriel et al., 1996, 2009; Latikka et al., 2001; Akhtari et al., 2016; Michel et al., 2017).

### Skin, Scalp and Skull

Skin, scalp, and the skull were built as a single layer with the shelling method used to obtain the brain phantom. But in this case, we used only PDMS as the constituent material because the electrical conductivities of these regions are low and similar to PDMS.

### Cerebrospinal Fluid

The gap between the brain and the skull is the CSF space. We filled this space with a saline solution that has an electrical conductivity similar to the CSF conductivity in human which is about 1.0–1.2 Sm^−1^.

### Implanted DBS Probe

DBS leads are comprised of four electrodes which lie at the site of stimulation, with four separate wires capable of delivering current to each contact. Each wire is wrapped in insulation to avoid interference with the other wires, and there is further insulation that comprises the entirety of the probe body. We used a commercial DBS lead (Medtronic 3387 lead) commonly used in DBS surgeries (Medtronic Lead Kit for DBS Stimulation, 2016).

The DBS probe was inserted into the conductive polymers during the solidification and through a guided opening on the brain phantom molds. After the solidification of the brain phantom, the molds were removed. We should note that we kept the stylet, supporting material, of the DBS probe to protect the integrity and structure of the wires inside the probe from damage. The DBS probes are very delicate and prone to damages as reported by the FDA (FDA, 2013). We believe that the presence of stylet has a negligible effect on the induced current in the lead wires as it is electrically isolated from the rest of the probe structure. The inside volume of the helical coils of the probe undergoes Faraday’s cage effect and hence the stylet will experience no induced electric field (Chapman et al., 2015).

**Figure 1:**
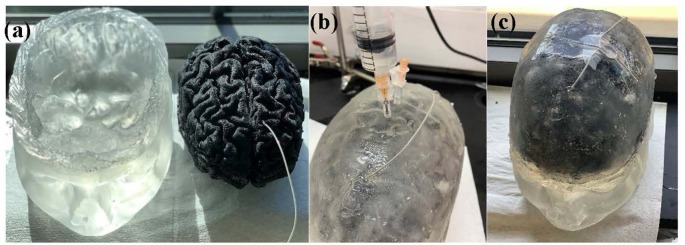
**(A)** Head model with skin, scalp and skull (left) and brain phantom with the implanted deep brain stimulation (DBS) probe (right). **(B)** Head phantom is enclosed and the saline solution that mimics the cerebrospinal fluid (CSF) is being injected into the head phantom. **(C)** Final realization of the anatomically accurate head phantom with the implanted DBS probe.

The final realization of the brain phantom that includes the realistic brain phantom and mimicked Cerebrospinal fluid (CSF) with implanted DBS probe in the hypothetical STN is seen in Figure 1C.

### Experimental Setup and Measurement of the Induced Current

**Figure 2:**
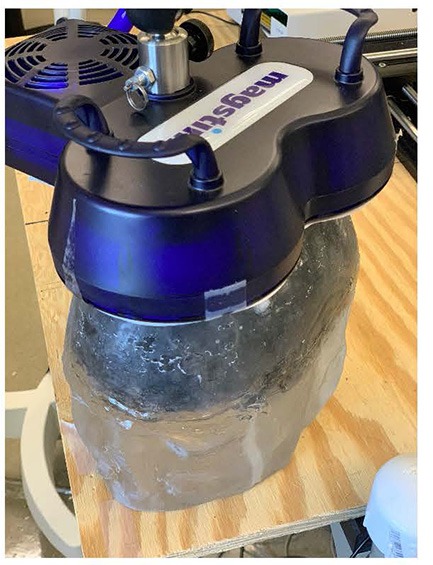
Experimental set-up where time-varying magnetic field by the transcranial magnetic stimulator is applied on the physical head phantom.

**Figure 3:**
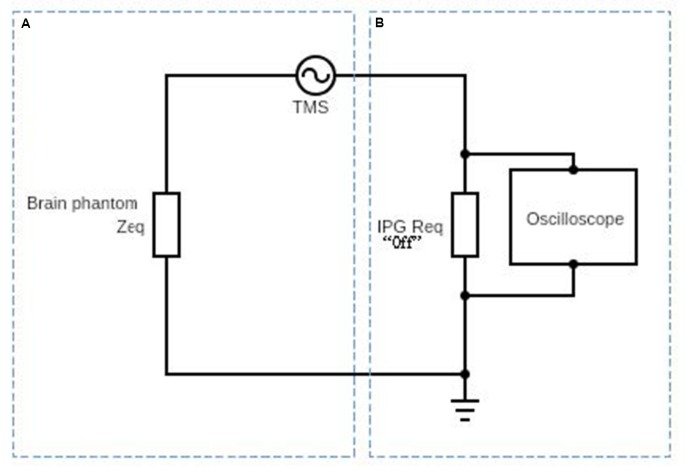
Schematic diagram af the circuit used to obtain the induced electric current on the DBS probe in the presence of the time-varyingmagnetic field. Part (**A**) represents the equivalent impedance *Z*_eq_ of the brain phantom. Part (**B**) represents the DBS probe and DBS pulse generator’s internal resistance and it is typically about 100 Ω (Deng et al., 2010).

An FDA-approved TMS device, Magstim (model: Rapid 2 with Magstim AirFilm coils) was used to apply TMS to the physical head phantom. TMS coils were placed about 1 cm on top of the DBS lead. The magnetic field was applied from 50% to 100% TMS coil’s current intensity with a single pulse and signal frequency of 2,500 Hz. The probe has one loop winding on top of the phantom. Then, we measured the induced voltage by measuring the voltage difference between the lead contacts and converted them into induced currents. The experimental set up is shown in Figure 2 and the circuital diagram is shown in Figure 3.

**Figure 4:**
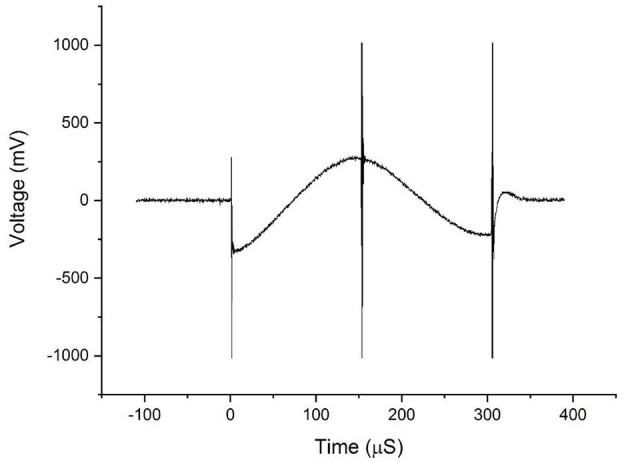
The waveforms obtained from the voltage measurements on the DBS probe during transcranial magnetic stimulation (TMS).

**Figure 5:**
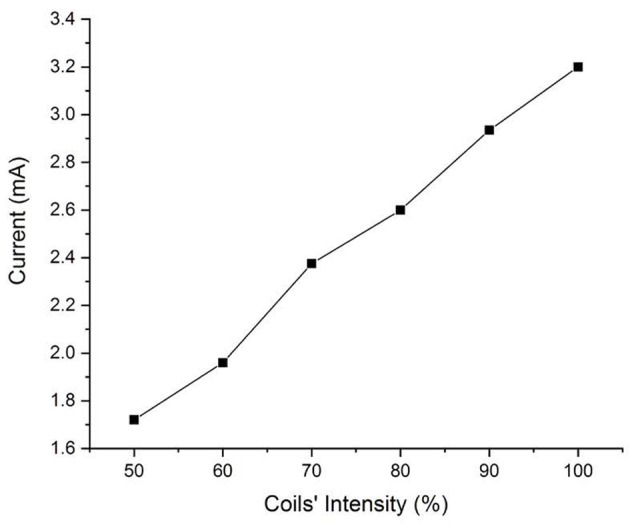
Induced currents with respect to TMS coil’s intensities.

In Figure 3, we show a schematic diagram of the circuit used to measure the induced voltage/current on the DBS. The circuit consists of two main parts A and B. Part A represents the equivalent impedance *Z*_eq_ of the brain phantom. In a real patient, this impedance would be the impedance of the neighboring regions of the inserted DBS leads. Part B represents the DBS probe and DBS pulse generator’s internal resistance and it is typically about 100 Ω (Deng et al., 2010). The resultant voltage waveform is shown in Figure 4 and the voltage values corresponding to coils’ intensities 50–100% are shown in Figure 5.

## Results

Figure 4 shows the bi-phasic waveforms obtained during the measurements. The waveform shown here corresponds to the measured induced voltage at 100% coils intensity. The waveform obtained with minor artifacts at the ends potentially due to an abrupt transition of the original pulse of the magnetic field and the artifact in the middle is a result of minor variations in the electric field due to switching off power transistor inside the TMS stimulator. Figure 5 shows the currents induced concerning changing the magnetic field strength produced by the TMS coils. The induced voltages are converted directly to induced current by dividing the voltage drop by the value of Req = 100 Ω.

## Discussion

From the results of the experiments on the physical head phantom, the induced current values are of the order of mA (1.71–3.20 mA), which are in the range of current values used in DBS (3.2–4.5 mA; Ramirez de Noriega et al., 2015). They are higher than values reported by Kumar et al. (1999) where they reported induced current in the range of 70–120 μA. Kühn and Huebl (2011) reported voltages in the rage of 0.2-2.8 V but indicated that the induced current duration was very short and did not reach the stimulation amplitude by the DBS therapy. However, Deng et al. (2010) reported higher values, in the range of 12.75–83 mA and (Shimojima et al., 2010) reported current density of 20 μC/cm^2^/phase which considered to be exceeding the safety threshold. Therefore, our measured values are consistent with Deng et al. (2010) and Shimojima et al. (2010). We theorize that the variation in the results may be due to several factors, the accuracy of the real DBS probe geometry, the medium in which the DBS probe is implanted, and the design and values of the electrical components of the complete circuit. We have experimentally measured the induced voltages and currents in the widely used Medtronic DBS lead. We accounted for the complexity of the brain anatomy and geometry. In our experimental model, we used our novel anatomically accurate brain and head phantom that mimics the impedance/conductivity of the brain as well as the CFS. Previous studies either lack geometrical accuracy of the medium in which the DBS probes were inserted in, DBS probe and lead design, or accurate impedance values. The geometry and impedance of the medium are important because they determine the magnitude of the electric field produced and in turn contribute to the current induced in the DBS probe. Such considerations will help to expand the study to include other DBS configurations and TMS coil orientations and placement. Thus, considering the prior work in the literature of TMS and DBS safety, our study is an improved analysis considering a more accurate brain phantom, actual DBS probe with accurate impedance. Previous studies showed that with the increased number of loops, induced current will increase promotionally. In this work we show that even with single loop, the current induced is on the range of unsafe limits. Moreover, Figure 4 shows that even at lower coil intensities, the induced current are noticeable and are in the mA range.

There are other sources of risk form such combination techniques like Lorentz forces. However, previous work by Shimojima et al. (2010) showed that there are no detectable movements on the DBS lead inserted in a gelatin phantom and therefore the risk from Lorentz forces is negligible. For that reason, and since our results show that it might be unsafe to combine DBS with TMS, we did not investigate other possible risks such as Lorentz forces in our study.

## Conclusion

In this work, we investigated the safety of combining DBS with TMS treatments. We developed an accurate physical model with commercially used DBS lead. Our measurements show that using a time-varying magnetic field applied by 100% TMS intensity in the presence of DBS will induce currents that are higher than the safe limits (3.2–4.5 mA) which may result in over-stimulation. These results are in an agreement with previous studies that considered the safety of the combination of TMS with DBS. We attempted to minimize the sources of errors that might result in higher variation in the result. We only used a simple configuration with one loop on the wire and used an accurate brain and head phantom that match the geometry and an averaged electrical conductivity. Our results suggest that even with the simplified setup, the level of induction is above the unsafe limits. Therefore, we recommend that the TMS should not be applied at 100% intensity directly over the implanted DBS leads in the STN area.

## Data Availability Statement

The datasets generated for this study are available on request to the corresponding author.

## Author Contributions

HM: manuscript drafting, created accurate and complex computational head models as well as the novel anatomically realistic brain and head phantom, and performed the experimental side of the research. FS: helped in manuscript drafting and evaluation. IC: assisted in the experiment and designed the circuit to acquire the induced current. KH: helped to conceive the research ideas and gave insightful inputs for the simulation and experimental parameters. RH: principal investigator, conceived the research idea, manuscript drafting, and evaluation.

## Conflict of Interest

The authors declare that the research was conducted in the absence of any commercial or financial relationships that could be construed as a potential conflict of interest.
